# Supervised consumption sites and crime: scrutinizing the methodological weaknesses and aberrant results of a government report in Alberta, Canada

**DOI:** 10.1186/s12954-020-00456-2

**Published:** 2021-01-06

**Authors:** James D. Livingston

**Affiliations:** Department of Criminology, Saint Mary’s University, 923 Robie Street, Halifax, NS B3H 3C3 Canada

**Keywords:** Supervised consumption site, Evaluation, Crime

## Abstract

To date, peer-reviewed research has found no evidence linking supervised consumptions sites (SCSs) to increased crime. Yet, in March 2020, a government Report released in the province of Alberta, Canada, presented the results of a review that reached a different conclusion. This commentary highlights the Report’s major methodological limitations with respect to its criminological components, including that crime was poorly operationalized and measured, change in crime was inadequately assessed, and the effect of SCSs on crime was not ascertained. It is argued that the magnitude of methodological flaws in the Report undermine the validity of its criminological claims and raise significant issues with the soundness of its conclusions.

## Background

Supervised Consumption Sites (SCSs) are harm reduction programs that offer a range of low-barrier services to people who use drugs, such as hygienic and supportive spaces for drug consumption, sterile drug using supplies, peer support, and ancillary health and social services. Major aims of SCSs include providing an environment for safer drug use, improving the health status of people who use drugs, and mitigating public disorder [1, 2].

Based on growing evidence of effectiveness and in the face of increasing numbers of opioid overdose deaths locally, seven SCSs and an overdose prevention site (OPS) were established in urban centres across the province of Alberta, Canada, from late 2017 to early 2019. Compared to SCSs, OPSs tend to be lower barrier, staffed and run by peers, less regulated, and offer a narrower range of clinical services [2, 3]. The SCSs were the subject of ongoing debate in Alberta leading up to the 2019 provincial election, which intensified upon the election of a conservative political party. Soon after, a Committee was appointed to examine the social and economic impacts of SCSs on local communities. The merit of SCSs as a harm reduction tool was deemed to be out of scope for the review [4], which expressly omitted potential benefits (e.g. reducing crime-related harms) from consideration. In March 2020, a Report was released describing the Committee’s findings [4]. The Report presents information across a range of indicators, including costs, needle debris, public disorder, opioid-related emergency incidents, drug-related deaths, and local business impacts. The Committee’s conclusions and recommendations, concentrating on what they perceived as “serious problems with supervised consumption and needle distribution” (p. 38), are also outlined. A central question considered in the Report, and the focus of this manuscript, was whether the SCSs exacerbated neighbourhood crime.

The Report generally concluded that crime increased around the SCSs—with the exception of one city—and inferred that the SCSs were the cause: “The preponderance of evidence provided by area residents and officials demonstrates that criminal activity near SCS has increased … SCS, therefore, are assumed to geographically concentrate the street-level drug market and other criminal activities” (p. 4) [4]. Other conclusions drawn by the Committee indicated that lawlessness existed around the SCSs, such as “de-policing” of neighbourhoods (p. iii) [4], reduced reporting of crimes to police, and worsened perceptions of public safety. The Report’s findings were characterized by Alberta’s Associate Minister of Mental Health and Addictions as “deeply troubling” and offering evidence of “a system of chaos … for communities around the sites” [5].

Soon after the Report’s release, a range of concerns about the review process were expressed by several groups and organizations [6]. An open letter endorsed by a multi-discipline collective of scholars and scientists, including the current author, called for the Report’s retraction due to major methodological flaws [7]. The current commentary describes three major limitations associated with the Report’s handling of crime-related data: crime was poorly operationalized and measured, change in crime was inadequately assessed, and the effect of SCSs on crime was not ascertained.

## Deficient measures of crime

Despite purporting to have measured “crime”, the Report actually assessed two indirect indicators of crime: police service calls and public perceptions of crime. Both indicators have inherent weaknesses that were exacerbated by the poor methodological quality of the review undertaken by the Committee.

The Report relied on police service call data which contains major limitations for validly representing the volume of crime and warrants special analytic and interpretive considerations. Police service calls are citizen- and police-initiated requests for police officers to attend incidents. A substantial proportion of police service calls involve unfounded allegations, uninvestigated incidents, and non-criminal matters (e.g. bylaw issues, security system alarms, traffic, medical event, and welfare checks), which appear to have been included in the data gathered by the Committee. Because a standard definition of “police service calls” was not employed, different police services provided different types of events in their data which distorted what was actually being measured. Consequently, “police service calls” broadly measured a variety of police activities as opposed to actual crimes. Despite this, the Report repeatedly misrepresented aggregated police service call data as “crime” data and erroneously equated changes in police service calls with changes in crime levels.

Many of the Report’s criminological claims were drawn from public perception data gathered from online surveys completed by a nonrepresentative sample. No steps were undertaken to identify or mitigate potential issues with survey sampling bias. Weighting was not performed to adjust for over- and under-represented groups. The Report did not mention whether controls were put in place to prevent multiple submissions by the same respondent. In addition to sampling issues, the psychometrics of the survey were unreported, so its reliability and validity are unknown. The survey asked respondents to recall their past experiences with numerous crime-related (and other) experiences before and after the opening of the SCSs, such as: “Prior to the supervised consumption services site opening in the area around your home, how often, if ever, did you see or experience people physically assaulting you or someone you live with?” Recall and recency bias are inherent limitations of such questions and of retrospective self-report surveys generally. Pre-existing beliefs and recent experiences distort people’s memory of events, and the accuracy of their recall decays over time. Such problems are particularly potent in criminology where a large body of literature demonstrates that people tend to misperceive and overestimate crime-related issues and trends [8]. The SCS review worsened these pre-existing limitations by asking respondents to pinpoint an event (the SCS opening) that took place up to two years prior to the survey administration and then recall specific incidents and patterns before and after that event. Such questions are prone to cognitive errors (e.g. telescoping) and, as such, rigorous victimization surveys have restricted recall to no more than six months, with extreme caution urged for extending the reference point beyond twelve months [9]. Several sources of error and bias in the survey were not appropriately identified or controlled which severely compromised the integrity of the resulting data and its use as a valid and reliable indicator of perceived crime.

The Report described gathering qualitative data from various forums (e.g. stakeholder meetings and town hall presentations) and an undisclosed number of written submissions. Numerous criminological claims repeated throughout the Report were drawn from this data—such as that SCSs “geographically concentrate” crime (p. 4) or that the police had abandoned the SCS neighbourhoods (p. 35)—without verification from other data sources. Numerous direct quotes drawn from this data source—almost entirely negative towards the SCSs—were presented in the Report, such as “the SCS is a lawless wasteland” (p. 25), “safety outside of the SCS is of concern” (p. 27), and “the [SCS] area is now used as a base of operations for property crime” (p. 29). The methods for recording and systematically analysing the qualitative data were not described in the Report. As such, the crime-related results and claims derived from this data source and presented in the Report should be viewed as very low quality, at high risk of bias, and neither credible nor dependable.

In sum, the Report employed a weak measure of crime because of the following issues: (a) improper handling of police service call data, (b) inadequate assessment of public perceptions of crime, and (c) inappropriate reliance on anecdotal accounts of crime.

## Inadequate assessment of change

In the Report, changes in crime were primarily assessed using a two-year percentage difference in police service calls in the area surrounding each SCS, such as: “calls for service increased by 18.1 percent between 2017 and 2018” (p. 15). The two-year period roughly spanned the year before and after the opening of each SCS. Methodological limitations and analytic flaws diminished the quality of the procedure used for assessing change. Subjective perceptions of changes in crime were also surveyed, but, as noted above, the quality of this data was very low.

The Report’s most significant flaw with respect to assessing change is the absence of statistical analyses. Descriptive data were displayed in tables and graphs to suggest changes in police service calls over time, but inferential statistics (e.g. bivariate or multivariate analysis, time series analysis, and spatial or proximity analyses) were not performed or reported. Rigorous statistical analysis is essential for answering the types of questions posed by the Committee. Without such statistical analysis, the size and significance of observed differences cannot be determined empirically, and the possibility of results being produced by chance, error, or confounding variables cannot be ruled out. Therefore, rather than objectively arriving at claims like “the amount of crime increased substantially in the area immediate to the SCS” (p. 14) [4], the Report relied on the subjective impressions to characterize the presence and magnitude of changes in police service calls which carried a serious risk of bias and error.

Claims of change were further weakened by the fact that the police service call data were not converted to rates. Standardizing crime data by the total population (typically expressed as a rate per 100,000 population) is the key for comparing crime-related trends over time, between groups, and across geographic areas. Failure to do so created uncertainty in the Report regarding the degree to which the observed changes in police service calls were impacted by population size variations [10]. This issue has particular relevance for the Report since different sized geographic units were compared. For instance, police service calls within a 250-m radius around the SCSs were compared with much larger areas (i.e. downtown, entire city). When presented as percentage differences, variations will naturally appear to be more dramatic in less populated areas with lower levels of crime (or police service calls) at baseline. A relatively small absolute increase in smaller areas (e.g. less populated, less crime) will translate into a relatively large percentage increase. For instance, in the city of Calgary, police service calls increased around the SCS by 541 calls (or 18.6% change) and also increased in the rest of the city by 11,300 calls (or 2.3% change). Focusing on the percentage difference rather than absolute numbers—on top of not standardizing the data—created a bias in the Report by systematically distorting the magnitude of changes within the small geographic units containing the SCS sites relative to the larger geographic units that served as comparison sites.

Police service calls were aggregated into annual counts and different call types were collapsed. As a result, the Report concealed important details regarding how police service calls changed. Monthly or quarterly data would have revealed variations occurring within the study period, allowing for the identification of relevant fluctuations, abrupt changes, anomalous events, and outliers in the data. Additionally, aggregating different types of police service calls did not permit an examination of how different calls (e.g. criminal or non-criminal) changed during the period of analysis. Disaggregated data were presented for one SCS but not the others, which raises an additional concern with biased and selective reporting of key outcomes in the Report.

The Committee assessed changes in police service calls over a two-year timeframe, roughly one year before and after the intervention (SCS opening). The short period of analysis hampered the meaningful assessment of crime-related changes by obscuring longer-term trends leading up and extending beyond the brief study period. It is possible that the changes observed within the two-year period were the product of crime-related trends occurring well before the study period. Additionally, the design was constrained to assessing immediate- and short-term effects of SCSs, preventing insights about the longer-term effects of SCSs from being revealed. Nowhere in the Report was it acknowledged that only short-term effects were considered, and appropriate caveats are not stated when describing the results and drawing conclusions.

In sum, the quality of the Report’s assessment of change in crime-related trends was severely diminished by several limitations: (a) not performing inferential statistical analysis, (b) failing to standardize police service call data, (c) aggregating police service call data, and (d) using a short period of analysis.

## Misattributing causation

The Report indicated that the SCSs were “responsible (at least in part)” (p. 3) for increasing crime [4]. However, the methods used to reach this conclusion, including the weak research design and absence of statistical analysis, precluded making such causal inferences. In fact, any observed effects could not be attributed specifically or partially to the SCSs using the approach described in the Report.

The review employed a pre- and post-observational design comparing police service calls in the areas adjacent to the SCSs with other geographic units (e.g. downtown, entire city). This design poses problems for establishing causality and, as such, confounding variables must be identified and adjusted for—either through experimental design or statistical analysis. Such steps were not undertaken and, consequently, Type I errors (i.e. false positives) run throughout the Report and falsely infer that the independent variable (SCSs) had a causal relationship with the dependent variable (crime). Non-SCS factors known to confound crime-related data include population demographics, neighbourhood characteristics, illegal drug markets, and police resources and operations (e.g. deployment levels, discretionary practices, and enforcement efforts) [1, 11]. Fluctuations in such extraneous variables during the pre- or post-study periods could produce changes in police service calls irrespective of the SCSs. Moreover, the Report compared police service calls in SCS neighbourhoods with that of larger geographic units (e.g. entire city) without establishing the comparability of those areas—an approach that is prone to produce misleading results especially because SCSs tend to be situated in distinctively marginalized neighbourhoods [10].

In sum, appropriate steps were not taken to isolate the effects of SCSs from extraneous variables, which undercuts all claims in the Report that inferred a causal connection between the SCSs and crime-related trends.

## Conclusion

Decision-makers deserve to have high-quality evidence about SCSs to help them make informed policy choices. The situation is dire as drug-related deaths continue to grow rapidly across North America. Urgent action is needed to effectively address the overdose epidemic. The problem with Alberta’s Report is not that it presented negative findings about SCSs, but rather that its results were produced using poor-quality evaluation methods and could be mistaken as credible evidence. Disseminating such unscrutinized results may lead decision-makers in the wrong direction during a time of crisis and thwart efforts to establish evidence-based harm reduction interventions, like SCSs.

Many of the Report’s findings are incongruent with the evidence contained with the body of peer-reviewed research examining the effectiveness of SCSs for almost two decades [1, 3, 12, 13]. For instance, the Report suggests that Alberta’s SCSs increased crime, but peer-reviewed studies have demonstrated different results in other jurisdictions, as was concluded recently in a systematic review: “There is no evidence that SCSs increase crime” (p. 2110) [3]. It is possible that Alberta’s SCSs are unique, but this is indiscernible from the Report. The magnitude of methodological flaws in the Report undermine the validity of its criminological claims and raise significant issues with the soundness of its conclusions. The Report’s authors neither acknowledged the methodological limitations sufficiently nor tempered their claims. Despite the inferences made in the Report, credible evidence establishing that SCSs exacerbate neighbourhood crime—in Alberta, Canada, or elsewhere—still does not exist.

## Data Availability

Not applicable.

## Abbreviations

SCS: Supervised consumption site
OPS: Overdose prevention site
